# Changes in heart rate variability parameters following pulsed-field ablation in patients with atrial fibrillation: A systematic review and meta-analysis HRV changes after pulsed-field ablation in AF

**DOI:** 10.1016/j.ijcha.2025.101766

**Published:** 2025-08-13

**Authors:** Xinyi Wang, Zhicheng Hu, Yan Yao, Pakezhati Maimaitijiang, Aiyue Chen, Lihui Zheng

**Affiliations:** Arrhythmia Center, Fuwai Hospital, National Center for Cardiovascular Diseases, Chinese Academy of Medical Sciences and Peking Union Medical College, Beijing, China

**Keywords:** Atrial fibrillation, Pulmonary vein isolation, Pulsed-field ablation, Heart rate variability, Cardiac autonomic function, Meta-analysis

## Abstract

**Introduction:**

Atrial fibrillation (AF) is a prevalent cardiac arrhythmia. Pulmonary vein isolation (PVI) is a cornerstone for catheter ablation for AF. Most recently, pulsed-field ablation (PFA) has emerged as a novel technique that minimizes tissue damage due to its tissue selectivity with promising clinical success. Despite its promise, the impact of PFA on cardiac autonomic function following PVI, particularly heart rate variability (HRV), and the differences between catheter ablation (CA) techniques, remain unclear.

**Methods:**

Following PRISMA guidelines, we conducted a systematic review and *meta*-analysis to evaluate changes in HRV parameters in three months post-catheter ablation using PFA, cryoballoon (CRYO), and radiofrequency (RF) ablation techniques. We searched databases including PubMed, Embase, Scopus, and Web of Science for relevant studies, focusing on HRV metrics such as SDNN, LF and HF in AF patients undergoing CA.

**Results:**

Our *meta*-analysis included 35 studies involving 6,267 AF patients. The results demonstrated a significant increase in heart rate and a decrease in HRV parameters such as SDNN and LF following CA in three months. Subgroup analyses revealed variations across ablation techniques, with PFA showing a milder impact on autonomic function compared to CRYO and RF. Notably, PFA was associated with a lower AF recurrence rate, suggesting superior therapeutic efficacy.

**Conclusion:**

PFA demonstrates a lower effect on cardiac autonomic function than the other two ablation techniques in three months. Notably, PFA showed a trend toward superior treatment outcomes, which may inform future choices of ablation technique for PVI in AF management. Further research is warranted to explore long-term autonomic effects and optimize treatment protocols.

## Highlights

• PFA has a milder impact on cardiac autonomic function, showing less reduction in HRV parameters compared to CRYO and RF ablation in three months.
• PFA is associated with a lower AF recurrence rate, indicating superior therapeutic efficacy.
• Understanding the differential impacts on autonomic function can guide personalized treatment choices, optimizing patient-specific therapeutic approaches.
• The higher efficacy of PFA in preventing AF recurrence supports its use as a primary ablation strategy.

## 1 Introduction

Atrial fibrillation (AF) is one of the most prevalent arrhythmias in clinical practice. AF not only severely impacts quality of life but also significantly increases the risk of stroke, heart failure, and other thromboembolic events [1]. Pulmonary vein isolation (PVI) has become a frontline treatment for AF to eliminate these abnormal signals and reduce AF episodes [2]. Current catheter ablation (CA) techniques include radiofrequency ablation (RF) and cryoballoon ablation (CRYO), with pulsed-field ablation (PFA) emerging as a highly studied method in recent years [3]. Unlike previous ablation techniques, PFA selectively ablates myocardial cells and minimizing damage to surrounding tissues with promising clinical success [4]. This approach reduces the incidence of complications and alleviates discomfort during the ablation procedure [5]. However, because numerous cardiac vagal intracardiac neurons are located around the pulmonary veins, ablation can inadvertently reduce cardiac vagal tone [6]. Such autonomic effects have been shown to correlate with a decreased risk of AF recurrence [7]. Although some evidence indicates that PVI significantly increases mean heart rate and markedly reduces parasympathetic activity, the specific impact of PFA and the effects of various ablation techniques on autonomic function remain unclear.

Heart rate variability (HRV) has emerged as the most widely utilised and valuable diagnostic tool to autonomic evaluation in clinical practice. It assesses cardiac autonomic activity by measuring the alterations in time intervals between consecutive heartbeats [8]. Studies have shown that a decrease in HRV signifies an impairment in the autonomic nervous system's capacity to preserve homeostasis within the body [9]. In cardiovascular diseases such as myocardial infarction and heart failure, for instance, this decline has been shown to portend a more unfavourable prognosis for the disease [10]. HRV can be divided into time-domain and frequency-domain metrics, including SDNN, SDANN, PNN50, LF, HF and LF/HF [11].

Several studies have investigated the association between different CA techniques and HRV values by examining the effects of performed PV ablation on cardiac autonomic function [[12], [13], [14], [15], [16]]. However, these studies have produced conflicting results due to differences in variables measured, sample sizes, and other factors. To date, no *meta*-analyses have supported a significant relationship between PFA and changes in HRV values and elucidated the differences between CA techniques. Thus, we conducted a *meta*-analysis to evaluate different changes in HRV values in 3 months following PFA, CRYO and RF.

## 2 Methods

This systematic review and *meta*-analysis were designed, conducted, and reported in accordance with the PRISMA (Preferred Reporting Items for Systematic Reviews and Meta-Analyses) guidelines [17].

### 2.1 Data sources and search strategy

Two investigators independently searched for relevant studies in electronic databases, including PubMed, Embase, Scopus, and Web of Science, using terms “pulmonary vein isolation” OR “ablation” OR “PVI” AND “Atrial fibrillation” AND “heart rate variability” OR “PNN50″ OR ”SDNN“ OR ”low frequency“ OR ”high frequency“ OR ”parasympathetic“ OR ”vagus“ OR ”sympathetic“ OR ”Deceleration Capacity“ OR ”DC“ OR ”SDANN“ OR ”LF“ OR ”HF“ OR ”VLF“ OR ”SDNNI“ OR ”Very low frequency“. Only studies published in English up to November 26, 2024, were included.

### 2.2 Eligibility criteria

Studies were included if they met the following criteria:

(1) Patients with AF receiving PFA, CRYO, or RF ablation.
(2) Quantitative analysis of each HRV parameter.
(3) Use of circumferential pulmonary vein isolation (CPVI) ablation procedures.
(4) Patients received no interventions other than CA.
(5) Before-and-after (pre-post) studies without a control group.

Exclusion criteria included:

(1) Animal studies, case reports, reviews, abstracts only, letters, conference proceedings, and *meta*-analyses.
(2) Studies providing insufficient data on any HRV parameters.
(3) Studies conducted on pediatric patients.

No restrictions were placed on sample sizes.

Additionally, in studies where PVI was combined with other methodologies, we extracted only the data related to HRV recordings specifically associated with PVI alone.

### 2.3 Data extracting and quality evaluation

Following a literature search, we evaluated the quality of the included studies using either the Newcastle–Ottawa Quality Assessment Scale for cohort studies tool or the Cochrane Risk of Bias tool for randomized controlled trials [18]. We documented the first author's name, year of publication, sample size, method of the ablation procedure, number of patients with paroxysmal atrial fibrillation and persistent atrial fibrillation, age of the patients, reported HRV parameters and recorded time points of HRV post-ablation. The mean and standard deviation (SD) outcomes were extracted. HRV parameters were analyzed in the time and frequency domains (Table 1).

**Table 1:** Descriptive characteristics of HRV parameters.

Acronym (unit)	Full name	Signification
Time domain		
SDNN (ms)	Standard Deviation of NN intervals	The standard deviation of normal-to-normal intervals is a general measure of HRV, reflecting the overall influence of the sympathetic and parasympathetic nervous systems on the heart.
SDANN (ms)	Standard Deviation of the Average NN intervals	The standard deviation of the average NN intervals calculated over 5-minute segments, known as SDANN, primarily reflects the variability in heart rhythm over longer periods.
pNN50(%)	Percentage of NN intervals that differ by more than 50 ms from the preceding interval	The proportion of adjacent NN intervals differing by more than 50 ms is a time-domain measure of heart rate variability, mainly reflecting parasympathetic nervous activity.
Frequency domain		
LF (ms^2^)	Power of low-frequency band (0.04–0.15H)	Low-frequency spectral power density, typically between 0.04 and 0.15 Hz, reflects the combined activity of the sympathetic and parasympathetic nervous systems and is commonly used to assess sympathetic nervous activity.
HF (ms^2^)	Power of high-frequency band (0.15–0.4 Hz)	High-frequency spectral power density, usually between 0.15 and 0.40 Hz, primarily reflects the influence of the parasympathetic nervous system on the heart.
LF/HF	LF/HF ratio	A higher LF/HF ratio generally indicates relatively strong sympathetic nervous activity, while a lower ratio indicates dominant parasympathetic nervous activity.

### 2.4 Statistical analysis

A *meta*-analysis was conducted to calculate the changes in HRV before and after ablation as effect size (ES), and all ES are reported as standard mean values (SMD). Due to the differences in follow-up time among different studies, the data included in this study are uniformly the follow-up results within three months, and the results beyond three months will be excluded. Cochran’s Q test was employed to assess statistical heterogeneity between studies, utilizing I^2^ statistics (where I^2^ = 0 % indicated no observed heterogeneity, and I^2^ ≥ 50 % suggested substantial heterogeneity) [19]. A random-effects model was applied when there was substantial variance (I^2^ > 50 % or p < 0.05); otherwise, a fixed-effect model was used. A sensitivity analysis was performed to evaluate the influence of individual studies on the ES. Publication bias was assessed using Begg’s and Egger’s regression asymmetry tests. The trim-and-fill method was applied to adjust the *meta*-analysis [20]. All *meta*-analyses were conducted using Stata/MP software, version 17.0. A p-value of < 0.05 was considered indicative of a significant statistical difference for all tests.

## 3 Results

### 3.1 Results of database search and study inclusion

**Fig. 1:**
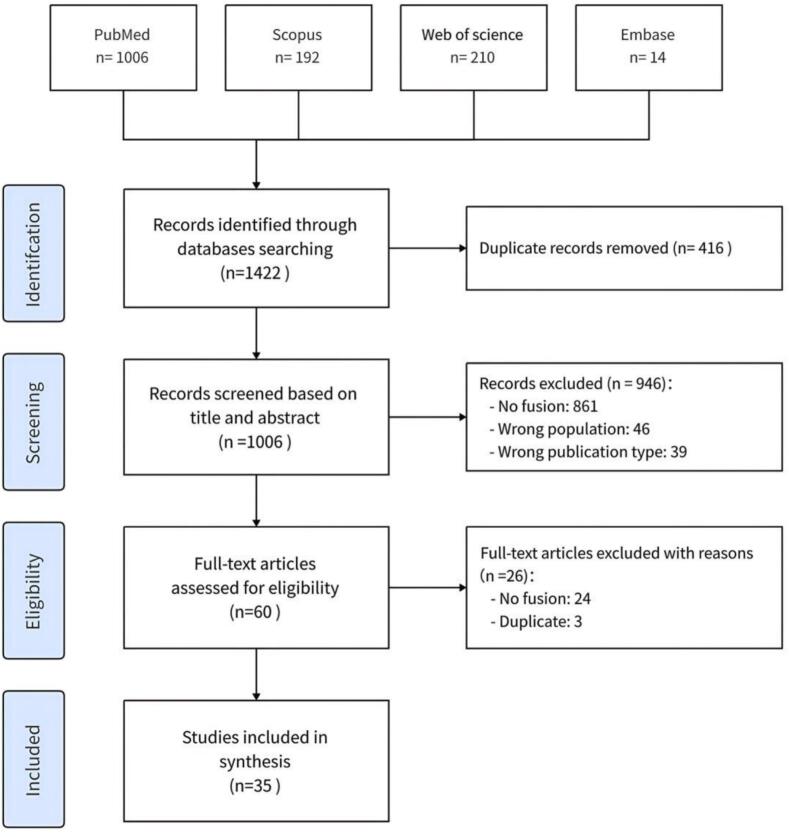
PRISMA flow diagram of the study selection process.

The process of literature search and selection is illustrated in Fig. 1 Our initial database search identified 1422 studies. After reviewing the titles and abstracts and removing duplicates, 416 studies were excluded. A detailed examination of the full texts of the remaining studies resulted in the exclusion of an additional 972 studies. Ultimately, 35 studies were included in our *meta*-analysis, encompassing 6,267 patients with AF who underwent PFA, CRYO, or RF ablation (Table 2).

**Table 2:** Literatures included in this study.

	Study	Study design	Country	Title	Sample Size	Paroxysmal AF n, (%)	Persistent AF n, (%)	Average age (year)	Ablation procedure	Recording time (post-ablation)	HRV measures
1	Andrade 2024 [12]	RCT-MC	Canada	Long-Term Differences in Autonomic Alterations After Cryoballoon vs Radiofrequency Atrial Fibrillation Ablation	346	346(100)	0(0)	58.8 ± 10.0	PVI/CRYO, RF	3–30 M	HR, SDANN
2	Zarębski 2024 [15]	PS-SC	Poland	Short-term deceleration capacity: a novel non-invasive indicator of parasympathetic activity in patients undergoing pulmonary vein isolation	24	24(100)	0(0)	54 ± 11	PVI/RF	1 D	HR, SDNN, RMMSD, pNN50, DC
3	Gerstenfeld 2024 [13]	RCT-MC	USA	Autonomic Effects of Pulsed Field vs Thermal Ablation for Treating Atrial Fibrillation: Subanalysis of ADVENT	379	379(3100)	0(0)	63.0 ± 8.3	PVI/PFA, CRYO, RF	3 M,6 M,12 M	HR, SDNN, SDANN
4	Valeriano 2024 [14]	PS-SC	Italy	Evaluating autonomic outcomes after pulmonary vein isolation: The differential effects of pulsed-field and radiofrequency energy	105	105(100)	0(0)	61.3 ± 9.8	PVI/PFA, RF	3 M	HR, SDNN, SDNNI, RMSSD
5	Iwakoshi 2024 [16]	PS-SC	Japan	Impact of Sleep Apnea on Nocturnal Parasympathetic Activity in Atrial Fibrillation Patients After Catheter Ablation − Implications for Heart Rate Variability Analysis	76	49 (64.5)	27 (35.5)	63.4 ± 11.6	PVI/CRYO, RF	3 M	SDNN, RMSSD, pNN50, HF, LF
6	Park 2023 [31]	PS-SC	Korea	Comparison of pulmonary vein isolation using cryoballoon, high-power short-duration, and conventional radiofrequency ablation for atrial fibrillation: a propensity score-weighted study	2647	NA	NA	60 (52–67)	PVI/CRYO, RF	3 M,12 M	HR, RMSSD, LF, HF, LF/HF
7	Liu 2023 [32]	PS-SC	China	Changes in Heart Rate after Pulmonary Vein Isolation in Patients with Paroxysmal Atrial Fibrillation and Sinus Bradycardia	91	91(100)	0(0)	58.71 ± 10.96	PVI/RF	2 D	HR, SDNN, SDANN, RMSSD, pNN50, LF/HF
8	Liao 2023 [33]	PS-SC	China	Fractal complexity alternations in paroxysmal atrial fibrillation patients with and without recurrence after pulmonary vein isolation	25	25(100)	0(0)	55.2 ± 8.7	PVI/CRYO, RF	1–3 M,6–12 M	HR, SDNN, RMSSD, pNN50, VLF, LF, HF, LF/HF
9	Suzuki 2023 [34]	RS-SC	Japan	Different time course effect of autonomic nervous modulation after cryoballoon and hotballoon catheter ablations for paroxysmal atrial fibrillation	190	190(190)	0(0)	64.5 ± 11.1	PVI/CRYO	1–12 M	HR,SDNN,LnLF,LnHF,LF/HF
10	Guo 2023 [26]	PS-SC	China	Effects of pulsed field ablation on autonomic nervous system in paroxysmal atrial fibrillation: A pilot study	18	18(100)	0(0)	65.8 ± 11.9	PVI/PFA	1 M	HR, SDNN, SDANN, RMSSD, pNN50, LF, HF, LF/HF
11	Chen 2022 [35]	PS-SC	China	Alterations of sympathetic dynamics after atrial fibrillation ablation by analysis sympathetic nerve activity provide prognostic value for recurrence and mechanistic insights into ablation	79	60(75)	19(25)	64 ± 15	PVI/CRYO, RF	3 M	HR, SKNA-frequency,SKNA-Amplitude
12	Sung 2022 [36]	PS-SC	China	Alteration of Skin Sympathetic Nerve Activity after Pulmonary Vein Isolation in Patients with Paroxysmal Atrial Fibrillation	37	37(100)	0(0)	58.9 ± 9.0	PVI/CRYO, RF	1 D,3 M	HR, SKNA-Amplitude
13	Stojadinović 2022 [37]	PS-SC	Czech Republic	Autonomic Changes Are More Durable After Radiofrequency Than Pulsed Electric Field Pulmonary Vein Ablation	31	NA	NA	53 ± 16	PVI/PFA, RF	1 D	HR
14	Mukai 2022 [38]	PS-SC	Japan	Effect of pulmonary vein isolation on the relationship between left atrial reverse remodeling and sympathetic nerve activity in patients with atrial fibrillation	22	18 (82)	4 (18)	64.6 ± 12.9	PVI/RF	3 M	HR, SKNA-frequency
15	Kiedrowicz 2022 [39]	PS-SC	Poland	Does a Vagal Response Indicate Cardiac Autonomic Modulation and Improve the Therapeutic Effect of Pulmonary Vein Isolation in Patients with Paroxysmal Atrial Fibrillation? Insights from Cryoballoon Ablation	296	296(100)	0(0)	60 (54–64)	PVI/CRYO	3, 6 and 12 M	HR, SDNN, SDNNI, SDANN, RMSSD, pNN50, LF, HF, LF/HF
16	Vraka 2022 [40]	PS-SC	Spain	The Dissimilar Impact in Atrial Substrate Modificationof Left and Right Pulmonary Veins Isolation after Catheter Ablation of Paroxysmal Atrial Fibrillation	61	61(100)	0(0)	NA	PVI/RF	1 D	SDNN, RMSSD
17	Musikantow 2022 [41]	RPS-SC	USA	Pulsed Field Ablation to Treat Atrial Fibrillation: Autonomic Nervous System Effects	120	120(100)	0(0)	59.1 ± 10.3	PVI/PFA, CRYO, RF	1 M	HR
18	Călburean 2021 [42]	PS-SC	Belgium	High vagal tone predicts pulmonary vein reconnection after cryoballoon ablation for paroxysmal atrial fibrillation	92	92(100)	0(0)	58 ± 13	PVI/CRYO	3 M	HR, RMSSD, SDANN, SDNNI, VLF, LF, HF, LF/HF
19	Saiz-Vivo 2021 [43]	PS-MC	Netherlands	Heart Rate Variability and Clinical Features as Predictors of Atrial Fibrillation Recurrence After Catheter Ablation: A Pilot Study	74	55(74)	19(26)	55.47 ± 12.79	PVI/RF	3 M	HR, RMSSD, SDNN
20	Tang 2021 [44]	PS-MC	Canada	Autonomic Alterations After Pulmonary Vein Isolation in the CIRCA-DOSE (Cryoballoon vs Irrigated Radiofrequency Catheter Ablation) Study	346	346(100)	0(0)	58.8 ± 10.0	PVI/CRYO, RF	3, 6, and 12 M	HR, SDANN
21	Olshausen 2021 [45]	PS-SC	Sweden	Sinus heart rate post pulmonary vein ablation and long-term risk of recurrences	482	353 (73)	129 (27)	59.3 ± 10.1	PVI/RF	1 D,3 M	HR
22	Călburean 2021* [46]	RS-SC	Belgium	High parasympathetic activity as reflected by deceleration capacity predicts atrial fibrillation recurrence after repeated catheter ablation procedure	110	72(65)	38(35)	64 (54–69)	PVI/RF	3 M	DC
23	Liu 2020 [47]	PS-SC	China	Amplitude reduction of autonomic nerve function is correlated with ablation lesion quality in patients with paroxysmal atrial fibrillation	72	72(100)	0(0)	58.2 ± 11.2	PVI/RF	3 D	SDNN,rMMSD,LnLF,LnHF
24	Marinković 2020 [48]	PS-SC	Serbia	A square root pattern of changes in heart rate variability during the first year after circumferential pulmonary vein isolation for paroxysmal atrial fibrillation and their relation with long–term arrhythmia recurrence	100	100(100)	0(0)	56 ± 11.2	PVI/RF	1 D,1 M,3 M,6 M,12 M	SDNN, RMSSD, HR, pNN50, LF/HF
25	Galloo 2019 [49]	PS-SC	Belgium	Impact of cryoballoon-guided pulmonary vein isolation on non-invasive autonomic tests in patients with paroxysmal atrial fibrillation	30	30(100)	0(0)	60.37 ± 9.02	PVI/CRYO	1 D,6M	HR
26	Styczkiewicz 2019 [50]	PS-SC	Italy	Cardiac autonomic regulation in patients undergoing pulmonary vein isolation for atrial fibrillation	20	5(25)	15(75)	61.3 ± 3.9	PVI/RF	1 M,6 M	HR, HF, LF, LF/HF
27	Jungen 2019 [51]	PS-SC	Germany	Respiratory sinus arrhythmia is reduced after pulmonary vein isolation in patients with paroxysmal atrial fibrillation	10	10(100)	0(0)	64 ± 3	PVI/CRYO, RF	2 D	HR, SDNN, RMSSD, pNN50, LF, HF, LF/HF
28	Cui 2019 [52]	PS-SC	Pennsylvania	Sympathetic responses induced by radiofrequency catheter ablation of atrial fibrillation	18	18(100)	0(0)	56 ± 3	PVI/RF	1 D	HR, RMSSD, SDNN, pNN50, HF, LF/HF, SKNA-frequency
29	Kanda 2018 [53]	PS-SC	Japan	Relation Between Autonomic Nervous Activity after Pulmonary Vein Isolation and Recurrence in Paroxysmal Atrial Fibrillation Patients	10	10(100)	0(0)	63 ± 8.5	PVI/CRYO, RF	3 and 6 M	HR, RMSSD, LF, HF, LF/HF, DC, AC
30	Vesela 2019 [54]	PS-SC	Czech Republic	Changes in heart rate variability in patients with atrial fibrillation after pulmonary vein isolation and ganglionated plexus ablation	26	19(73)	7(27)	61 ± 11	PVI/RF	1 D	HR, SDNN, LF, HF, LF/HF
31	Chen 2018 [55]	PS-SC	China	Low heart deceleration capacity imply higher atrial fibrillation-free rate after ablation	154	154(100)	0(0)	60.0 ± 10.1	PVI/RF	3 D,3M,6M	DC, AC, HR, SDNN, RMSSD, LF/HF
32	Yanagisawa 2018 [56]	RS-SC	Japan	Assessment of autonomic nervous system modulation after novel catheter ablation techniques for atrial fibrillation using multiple short-term electrocardiogram recordings	65	65(100)	0(0)	62.5 ± 9.2	PVI/CRYO	1, 3, 6, and 12 M	HR,SDNN,LnLF,LnHF,LF/HF
33	Mori 2018 [57]	RS-SC	Japan	Analysis of the heart rate variability during cryoballoon ablation of atrial fibrillation	25	25(100)	0(0)	67.0 (60.8–69.3)	PVI/CRYO	1 D	LF, HF, LF/HF
34	Kuyumcu 2017 [58]	PS-SC	Turkey	The short-term impact of the catheter ablation on noninvasive autonomic nervous system parameters in patients with paroxysmal atrial fibrillation	45	45(100)	0(0)	46 ± 10	PVI/CRYO	1 M,3 M	HR
35	Yanagisawa 2017 [59]	RS-SC	Japan	Vagal response in cryoballoon ablation of atrial fibrillation and autonomic nervous system: Utility of epicardial adipose tissue location	41	41(100)		65.6 ± 10.8	PVI/CRYO	1D,1M	HR,SDNN,RMSSD,Ln LF,Ln HF,LF/HF

HR: Heart rate; SDNN: Standard Deviation of NN intervals; SDANN: Standard Deviation of the Average NN intervals; pNN50(%): Percentage of NN intervals that differ by more than 50 ms from the preceding interval; LF: Power of low-frequency band; HF: Power of high-frequency band; LF/HF: LF/HF ratio.

### 3.2 Main effects analysis

#### 3.2.1 Heart rate (HR)

A total of 2,077 effect size calculations from 23 studies were analyzed. The *meta*-analysis demonstrated a significant increase in HR following CA (ES = 7.526 (6.044, 9.008), p < 0.001) (Table 3).

**Table 3:** Catheter ablation and HRV parameters.

HRV parameters	Effect size	No. of effect sizes	Pre–post mean difference	p-value	Between studies I^2^(heterogeneity), p-value	Meta-regression P>|t|
Time domain						
HR	2077	23	7.526(6.044,9.008)	**＜0.001**	78.9 %,＜0.001	＜0.001
SDNN	978	15	−18.806(–23.479, −14.133)	**＜0.001**	48.3 %,0.01	＜0.001
SDANN	982	5	−19.049(−19.752, −18.346)	**＜0.001**	68.8 %,0.002	＜0.001
pNN50%	130	6	−3.616(−7.297,0.065)	0.054	96.4 %, <0.001	＜0.001
Frequency domain						
LF	528	7	−243.065(−371.205, −114.926)	**<0.001**	97.6 %, <0.001	＜0.001
HF	476	7	−55.348(−84.955, −25.741)	**<0.001**	91.0 %, <0.001	＜0.001
LF/HF	700	17	−0.324(−0.461, −0.187)	**<0.001**	82.0 %, <0.001	＜0.001

AF: Atrial fibrillation; HR: Heart rate; SDNN: Standard Deviation of NN intervals; SDANN: Standard Deviation of the Average NN intervals; pNN50(%): Percentage of NN intervals that differ by more than 50 ms from the preceding interval; LF: Power of low-frequency band; HF: Power of high-frequency band; LF/HF: LF/HF ratio.

#### 3.2.2 SDNN

The *meta*-analysis of 978 effect size calculations of 15 studies revealed a significant decrease following CA in parameters of SDNN (ES = −18.806(–23.479, −14.133), p < 0.001) (Table 3).

#### 3.2.3 SDANN

There was a decrease in SDANN in 982 effect size calculations from 5 studies following CA compared to pre-treatment (ES = −19.049 (−19.752, −18.346), p < 0.001) (Table 3).

#### 3.2.4 pNN50%

From 130 effect size calculations across 6 studies, a slight, non-significant decrease in pNN50% was observed after CA (ES = −3.616(−7.297, 0.065), p = 0.054) (Table 3).

#### 3.2.5 LF

Out of the 7 studies, 528 effect sizes were calculated. LF declined significantly following CA (ES = −243.065(−371.205, −114.926), p < 0.001) (Table 3).

#### 3.2.6 HF

Similarly, 528 effect sizes from 7 studies showed a significant reduction in HF values after CA (ES = −55.348(−84.955, −25.741), p < 0.001) (Table 3).

#### 3.2.7 LF/HF ratio

Based on 700 effect sizes from 17 studies, the results demonstrated a significant change in the LF/HF ratio following CA (ES = −0.324 (−0.461, −0.187), p < 0.001) (Table 3).

We performed subgroup analyses of HRV parameters categorized by the ablation procedure.

#### 3.2.8 Heart Rate (HR)

Subgroup analyses according to PFA, CRYO and RF ablation procedures were performed by 300 effect size calculations from 4 studies for PFA,1096 effect size from 13 studies for CRYO and 2117 effect size calculations from 14 studies for RF. The results demonstrated a significant increase in HR after CA in ablation procedures of PFA (ES = 5.494(3.448,7.540), p < 0.001), CRYO (ES = 9.730(7.769,11.691), p < 0.001), and RF (ES = 5.831(4.132, 7.529), p < 0.001) (Table 4).

**Table 4:** Summary of mean difference (MD) estimates (95% confidence intervals (CI)) association between catheter ablation and HRV parameters by the ablation procedure.

Subgroup	No. of studies	No. of effect sizes	Summary MD	P-value	Between studies I2(P heterogeneity)	Between subgroups Q (P heterogeneity)
HR						
PFA	4	300	5.494(3.448,7.540)	**<0.001**	13.7 % (0.324)	62.61(<0.001)
CRYO	13	1096	9.730(7.769,11.691)	**<0.001**	73.5 % (<0.001)	
RF	14	2117	5.831(4.132, 7.529)	**<0.001**	57.8 % (0.004)	
SDNN						
PFA	2	81	−11.027(−19.136, −2.917)	**<0.001**	0.0 % (0.463)	2.44(0.295)
CRYO	7	452	−17.496(−19.707, −15.285)	**<0.001**	58.0 % (0.027)	
RF	10	491	−17.684(−19.872, −15.495)	**<0.001**	48.6 % (0.041)	
SDANN						
PFA	2	81	−5.878(−14.611,2.856)	0.187	0.0 % (0.729)	9.00(0.011)
CRYO	4	448	−17.238(–22.606, −11.870)	**<0.001**	65.6 % (0.033)	
RF	2	799	−15.797(−24.270, −7.324)	**<0.001**	78.1 % (0.033)	
pNN50%						
PFA	1	16	0.900(−0.218,2.018)	0.115		127.73(<0.001)
CRYO	2	60	−1.077(−2.934,0.781)	0.256	57.8 % (0.124)	
RF	7	52	−7.848(−14.844, −0.852)	0.394	78.3 % (0.010)	
LF						
PFA	1	18	3.700(1.390,6.010)	0.515		327.16(<0.001)
CRYO	5	439	−242.182(−274.797, −209.567)	**<0.001**	57.5 % (0.052)	
RF	3	107	−276.476(−318.660, −234.292)	**<0.001**	13.5 % (0.315)	
HF						
PFA	1	18	4.200(2.893,5.507)	0.474		74.80(<0.001)
CRYO	5	458	−57.439(−67.902, −46.977)	**<0.001**	0.0 % (0.585)	
RF	2	35	−71.947(−92.388, −51.505)	**<0.001**	0.0 % (0.778)	
LF/HF						
PFA	1	18	−0.300(−6.963,6.363)	0.231		25.92(<0.001)
CRYO	10	652	−0.227(−0.397, −0.057)	**<0.001**	76.3 % (0.002)	
RF	9	606	−0.432(−0.579, −0.284)	**<0.001**	58.9 % (0.063)	

PFA: Pulse-field ablation; CRYO: Cryoballoon ablation; RF: Radiofrequency ablation; HR: Heart rate; SDNN: Standard Deviation of NN intervals; SDANN: Standard Deviation of the Average NN intervals; pNN50(%): Percentage of NN intervals that differ by more than 50 ms from the preceding interval; LF: Power of low-frequency band; HF: Power of high-frequency band; LF/HF: LF/HF ratio.

#### 3.2.9 SDNN

For SDNN, subgroup analyses according to PFA, CRYO and RF ablation procedures were performed by 81 effect size calculations from 2 studies for PFA,452 effect size from 7 studies for CRYO and 491 effect size calculations from 10 studies for RF. The results indicated a significant decrease in SDNN after CA in ablation procedures of PFA (ES = -11.027(−19.136, −2.917), p < 0.001), CRYO (ES = −17.496(−19.707, −15.285), p < 0.001), and RF (ES = −17.684(−19.872, −15.495), p < 0.001) (Table 4).

#### 3.2.10 SDANN

Subgroup analyses according to PFA, CRYO and RF ablation procedures were performed by 81 effect size calculations from 2 studies for PFA,448 effect size from 4 studies for CRYO and 799 effect size calculations from 2 studies for RF. The results indicated a significant decrease in SDANN after CA in ablation procedures of CRYO (ES = −17.238(–22.606, −11.870), p < 0.001), and RF (ES = −15.797(−24.270, −7.324), p < 0.001), but not for PFA (ES = -5.878(−14.611,2.856), p = 0.187) (Table 4).

#### 3.2.11 pNN50%

Calculations of 16 effect sizes from 1 study for the PFA, 60 effect size from 2 studies for CRYO and 52 effect size calculations from 7 studies for RF were used to perform subgroup analyses based on CA procedures. The results did not indicate a significant decrease in SDNN after CA in ablation procedures of PFA (0.900(−0.218,2.018), p = 0.115), CRYO (ES = −1.077(−2.934,0.781), p = 0,256), and RF (ES = −7.848(−14.844, −0.852), p = 0.394) (Table 4).

#### 3.2.12 LF

Considering the ablation procedure, subgroup analyses were conducted using 18 effect sizes from 1 study for the PFA, 439 effect size from 5 studies for CRYO and 107 effect size calculations from 3 studies for RF. In both CRYO (ES = −242.182(−274.797, −209.567), p < 0.001) and RF (ES = -276.476(−318.660, −234.292), p < 0.001) ablation procedures, the results showed a significant reduction in LF following CA, but not for PFA (ES = − 3.700(1.390,6.010), p = 0.515) (Table 4).

#### 3.2.13 HF

Subgroup analyses according to PFA, CRYO and RF ablation procedures were performed by 81 effect size calculations from 2 studies for PFA,458 effect size from 5 studies for CRYO and 35 effect size calculations from 2 studies for RF. The results indicated a significant decrease in HF after CA in ablation procedures of CRYO (ES = −57.439(−67.902, −46.977), p < 0.001), and RF (ES = −71.947(−92.388, −51.505), p < 0.001), but not for PFA (ES = 4.200(2.893,5.507), p = 0.474) (Table 4).

#### 3.2.14 LF/HF

The results of the subgroup analyses of ablation procedures based on 18 effect size calculations from 1 study for PFA,652 effect size from 10 studies for CRYO and 606 effect size calculations from 9 studies for RF indicated a significant increase in LF/HF following CRYO and RF ablation (ES = -0.227(−0.397,-0.057), p < 0.001; ES = −0.432(−0.579,-0.284), p < 0.001). However, no LF/HF value change was observed after PFA ablation (ES = -0.300(−6.963,6.363), p = 0.231) (Table 4).

We further analyzed AF recurrence in the included studies based on the proportion of recurrences in the included AF population. Based on 13 studies of 3083 effect sizes, the overall post-CA AF recurrence rate was 0.212 (0.204,0.220). According to this average recurrence level, all included studies were divided into low recurrence and high recurrence groups to compare the differences in HRV between the two groups. Subgroup analyses according to PFA, CRYO, and RF ablation procedures were performed using 38 effect size calculations from 2 studies for PFA,532 effect size calculations from 5 studies for CRYO, and 2513 effect size calculations from 7 studies for RF. The results indicated profound differences between 3 different CAs with lower recurrence in the PFA subgroup. (PFA vs CRYO vs RF:0.059(0.038,0.080), 0.221(0.197,0.244), 0.242(0.232,0.251)) (Table 5).

**Table 5:** Summary of mean difference (MD) estimates (95% confidence intervals (CI)) association between RF ablation and HRV parameters by recurrence rates.

Subgroup	No. of studies	No. of effect sizes	Summary MD	P-value	Between studies I2(P heterogeneity)
HR					
Low recurrence	2	71	13.51(10.97,16.05)	**<0.001**	<50 % (0.893)
High recurrence	8	1341	6.40(3.64,10.21)		97.4 % (<0.001)
SDNN					
Low recurrence	2	113	−30.37(−37.33, –23.42)	**<0.001**	<50 % (0.634)
High recurrence	3	189	–22.98(−30.43, −15.54)		62.1 % (0.022)
LF/HF					
Low recurrence	1	41	−0.07(−0.17,0.03)	**<0.001**	<50 % (0.348)
High recurrence	5	2904	−0.33(−0.44, −0.23)		91.0 % (<0.001)

HR: Heart rate; SDNN: Standard Deviation of NN intervals; SDANN: Standard Deviation of the Average NN intervals; pNN50(%): Percentage of NN intervals that differ by more than 50 ms from the preceding interval; LF: Power of low-frequency band; HF: Power of high-frequency band; LF/HF: LF/HF ratio.

In addition, we further divided the included studies into low-recurrence and high-recurrence subgroups based on the level of relapse rate to compare the differences in the altered values of HRV values between the subgroups and thus analyze the potential relationship between the degree of altered HRV values and relapse. Among all included HRV metrics, the degree of HR, SDNN, and LF/HF alteration differed significantly between the two groups.HR (13.51 ± 2.54 vs. 6.40 ± 2.77) and SDNN (−30.37 ± 6.96 vs. −17.66 ± 7.89) showed greater values of alteration in the low-recurrence group, whereas the degree of alteration of LF/HF showed greater (−0.41 ± 0.07 vs. −0.07 ± 0.1) (Table 5).

Publication Bias Assessment.

**Fig. 2:**
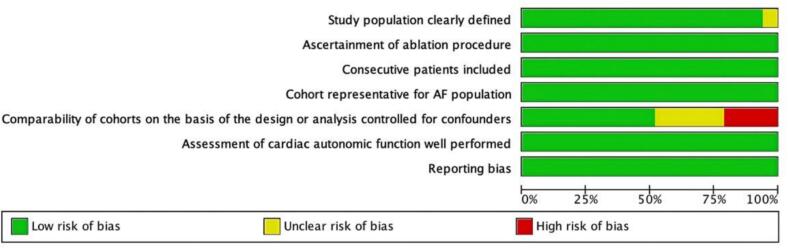
Methodological quality graph for the risk of bias from included studies using the Newcastle–Ottawa Quality Assessment Scale for Cohort Studies tool.

**Fig. 3:**
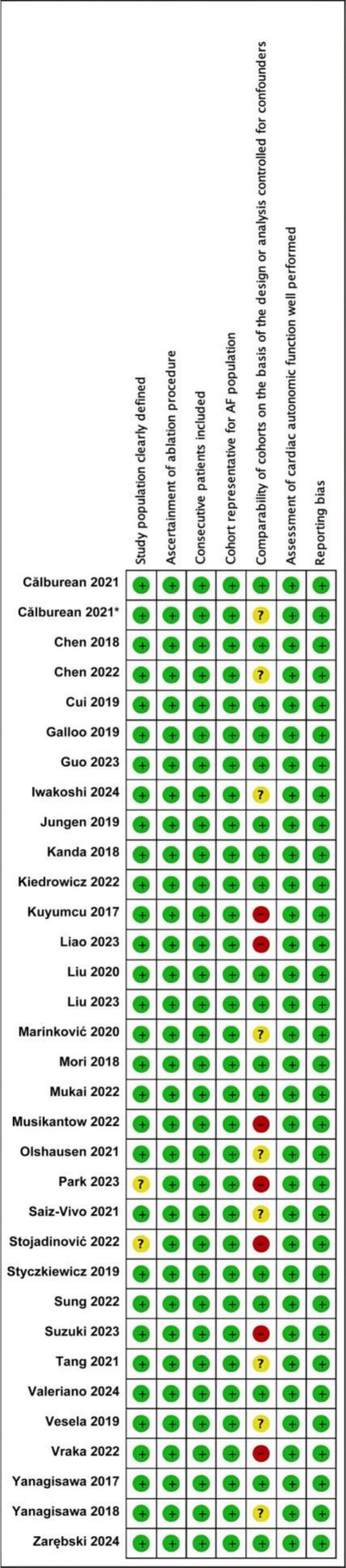
Methodological quality summary for the risk of bias from the included studies using the Newcastle–Ottawa Quality Assessment Scale for Cohort Studies.

**Fig. 4:**
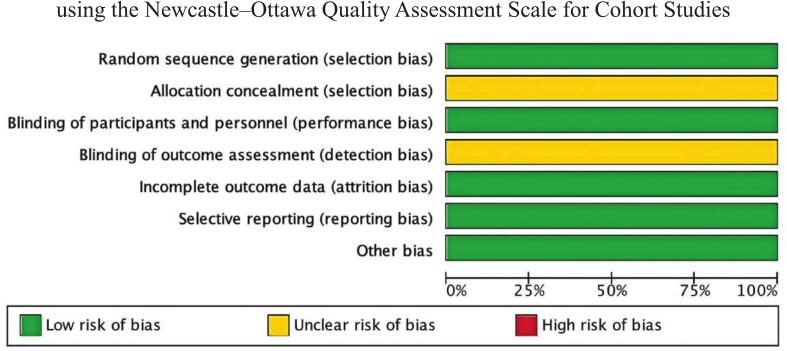
Methodological quality graph for the risk of bias from included studies using the Cochrane Risk of Bias tool for Randomized Controlled Trials.

**Fig. 5:**
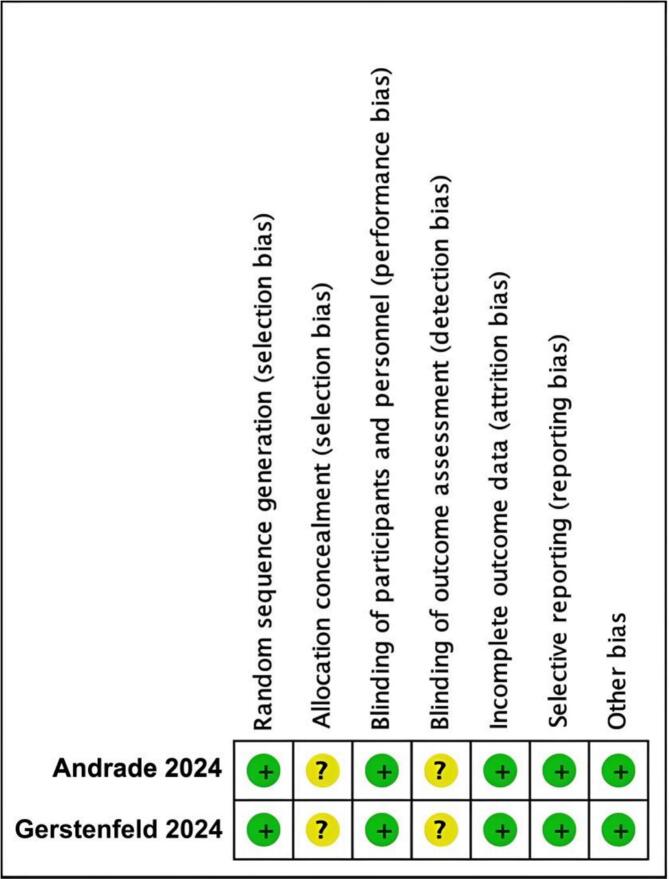
Methodological quality summary for the risk of bias from the included studies using the Cochrane Risk of Bias tool for Randomized Controlled Trials.

The funnel plots for visual inspection of the bias are reported, and in Fig. 2, Fig. 3, Fig. 4, Fig. 5, there was no significant bias.

## 4 Discussion

### 4.1 Major findings

**Fig. 6:**
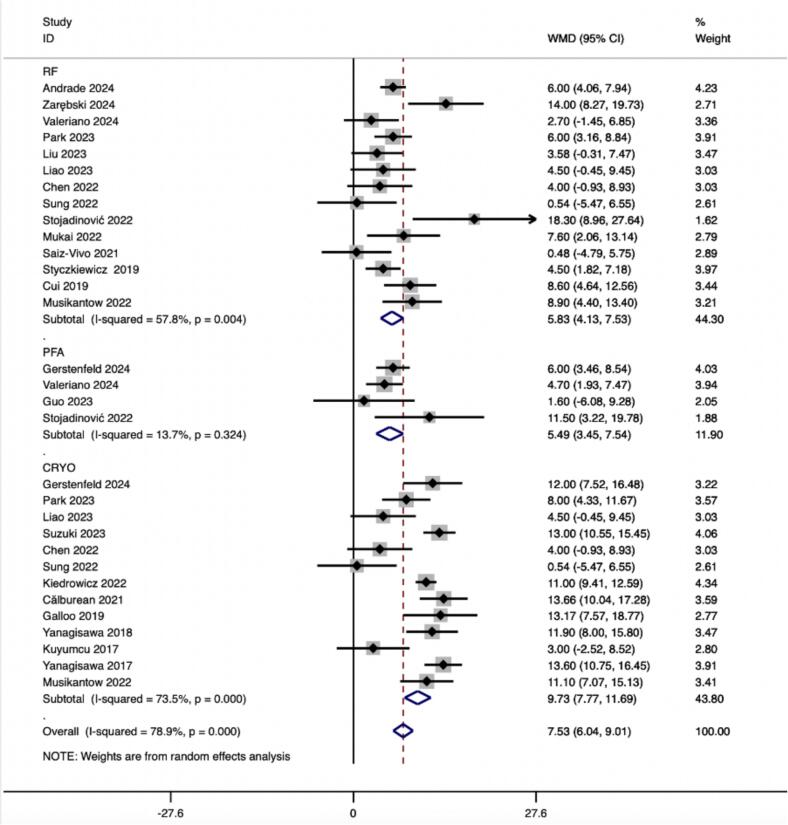
Forest plot of HR.

**Fig. 7:**
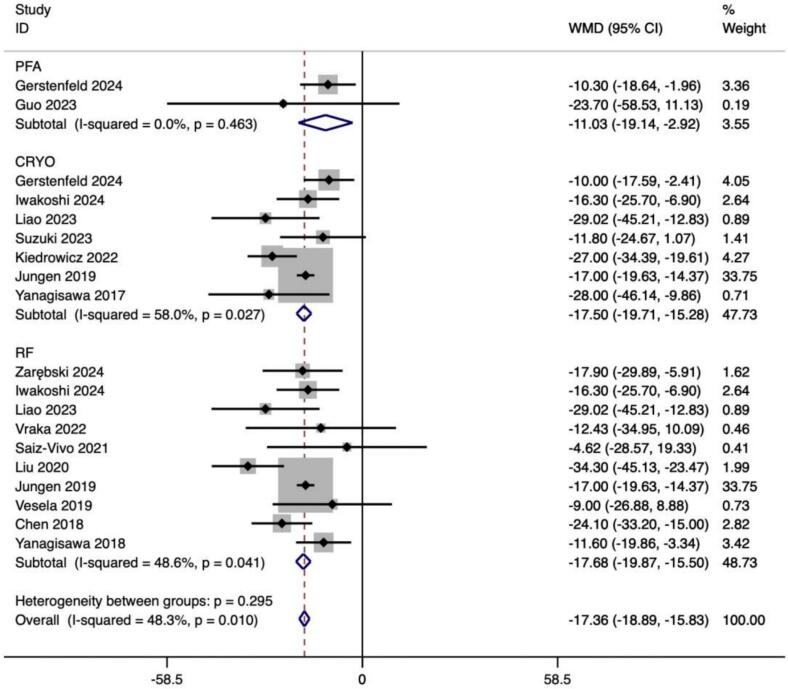
Forest plot of SDNN.

**Fig. 8:**
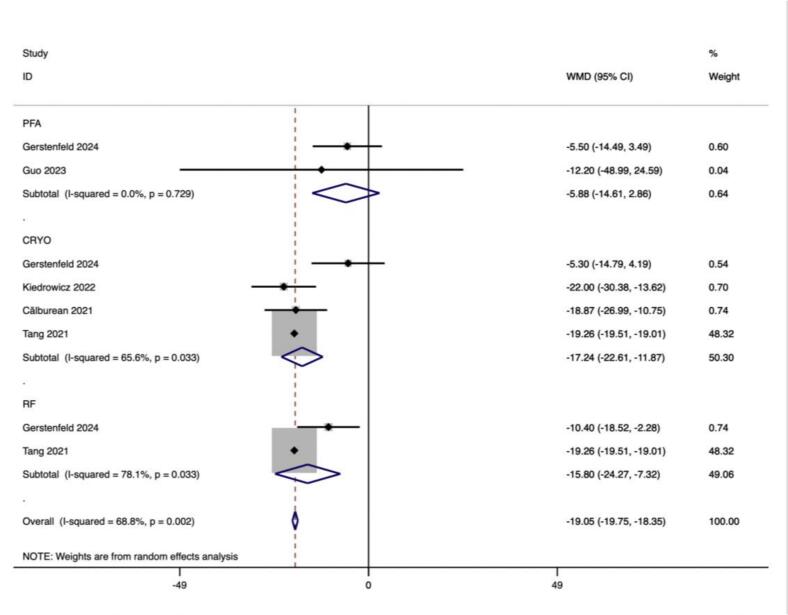
Forest plot of SDANN.

**Fig. 9:**
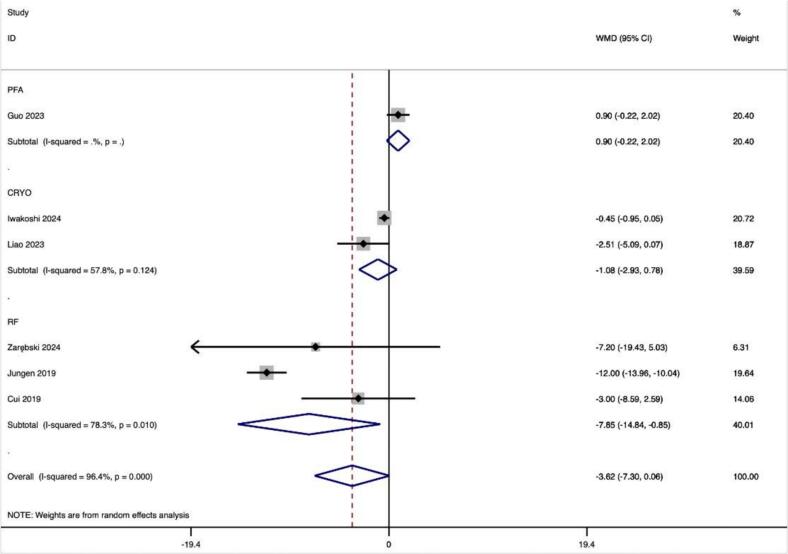
Forest plot of pNN50%.

**Fig. 10:**
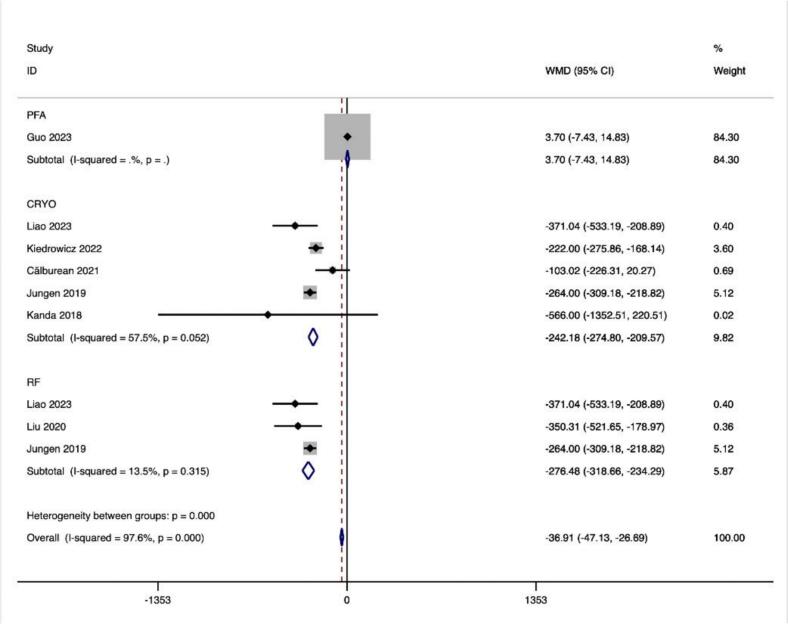
Forest plot of LF.

**Fig. 11:**
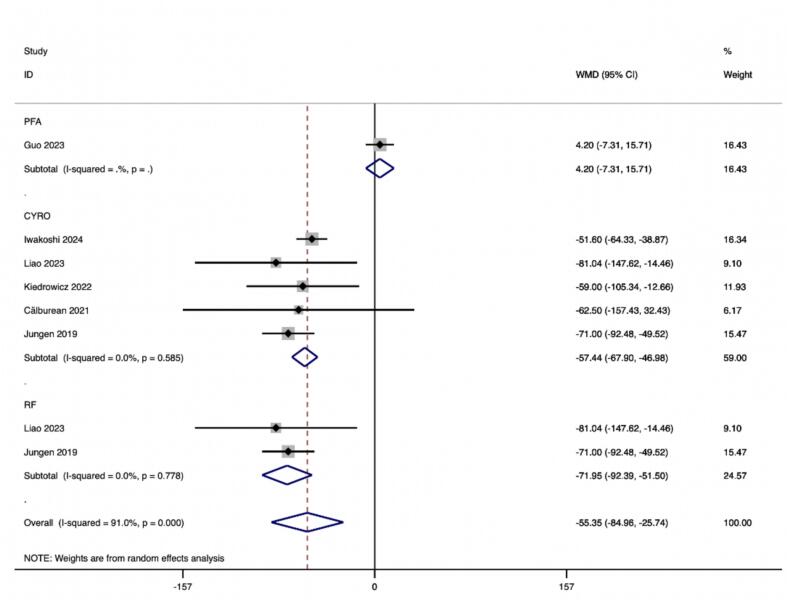
Forest plot of HF.

**Fig. 12:**
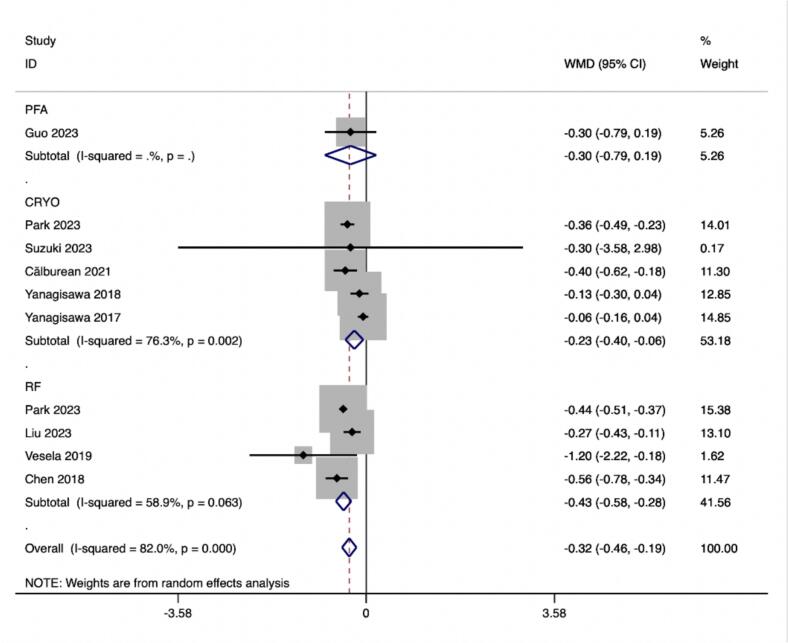
Forest plot of LF/HF.

**Fig. 13:**
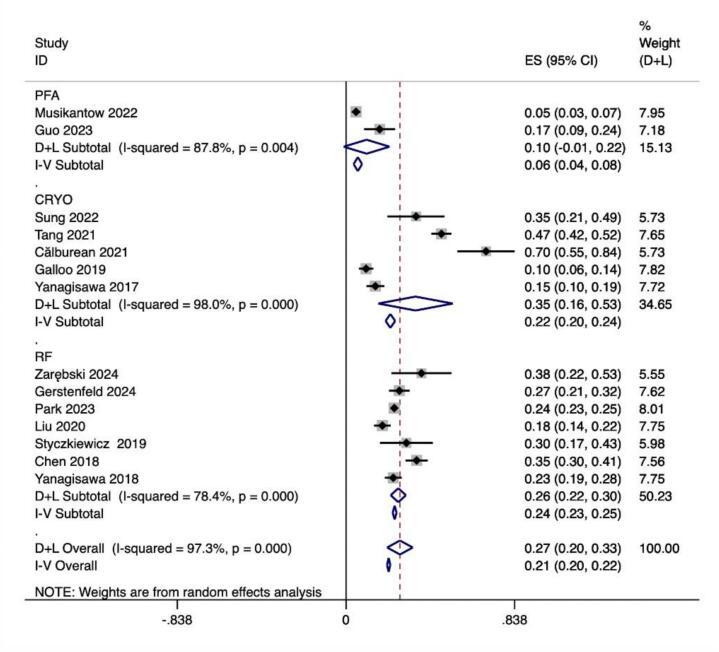
Forest plot of recurrence rate.

**Fig. 14:**
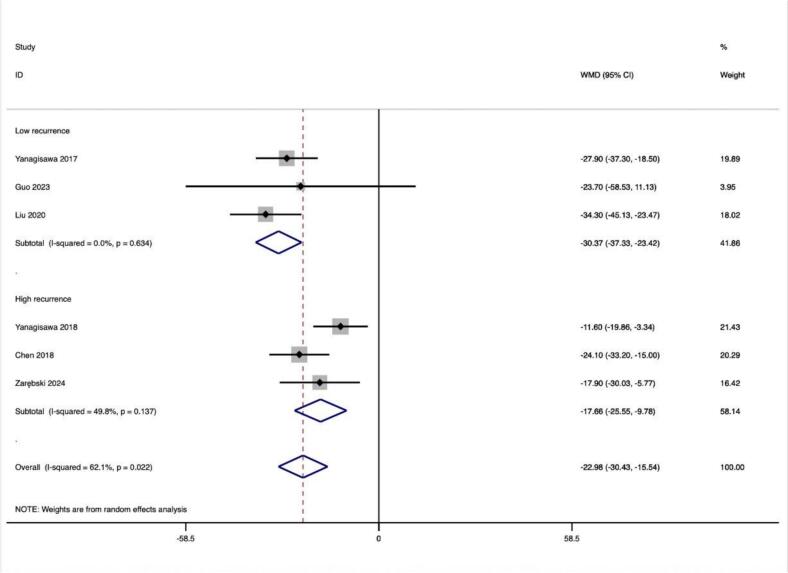
Forest plot of HR in the low recurrence rate group.

**Fig. 15:**
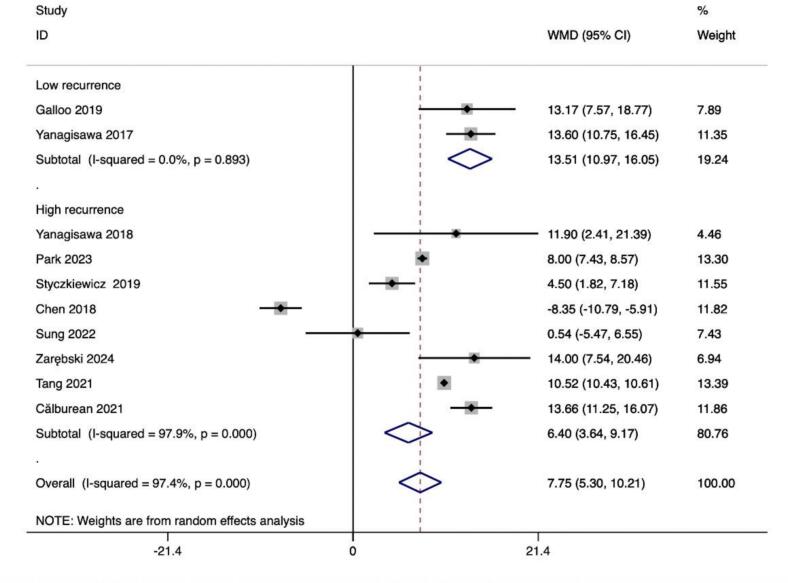
Forest plot of SDNN in the low recurrence rate group.

**Fig. 16:**
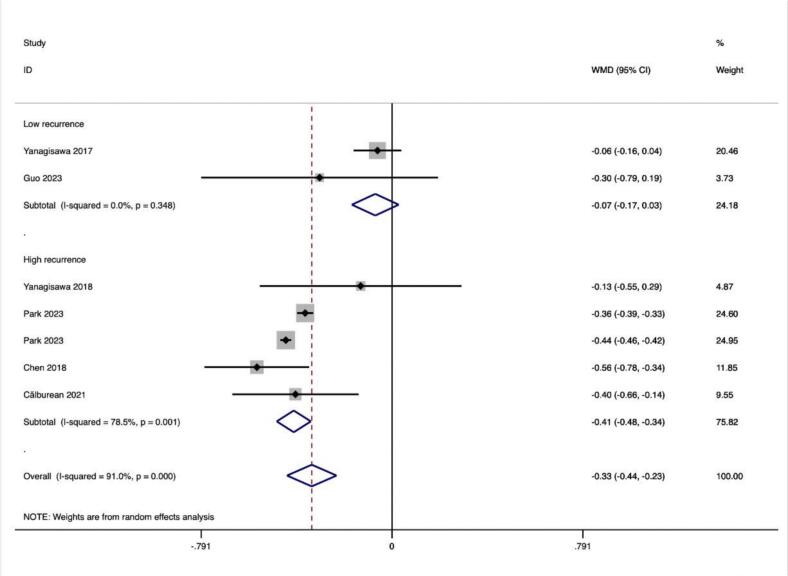
Forest plot of LF/HF in the low recurrence rate group.

**Fig. 17:**
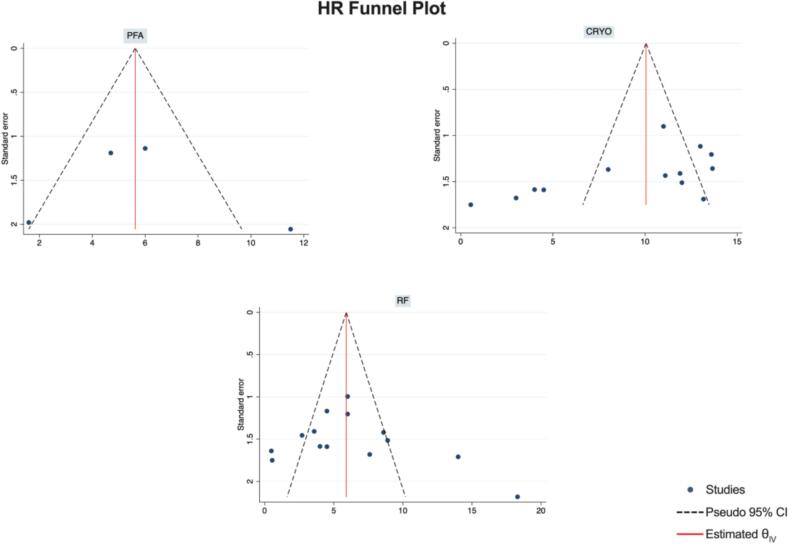
Funnel plots of HR.

**Fig. 18:**
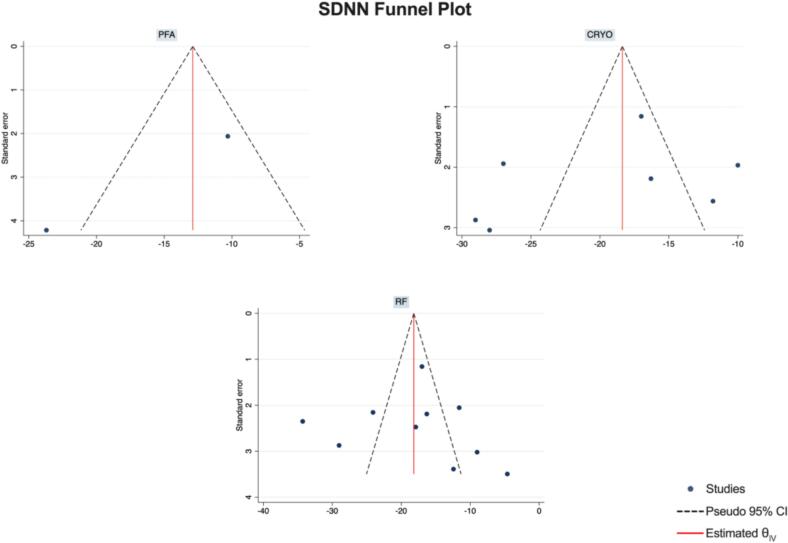
Funnel plots of SDNN.

**Fig. 19:**
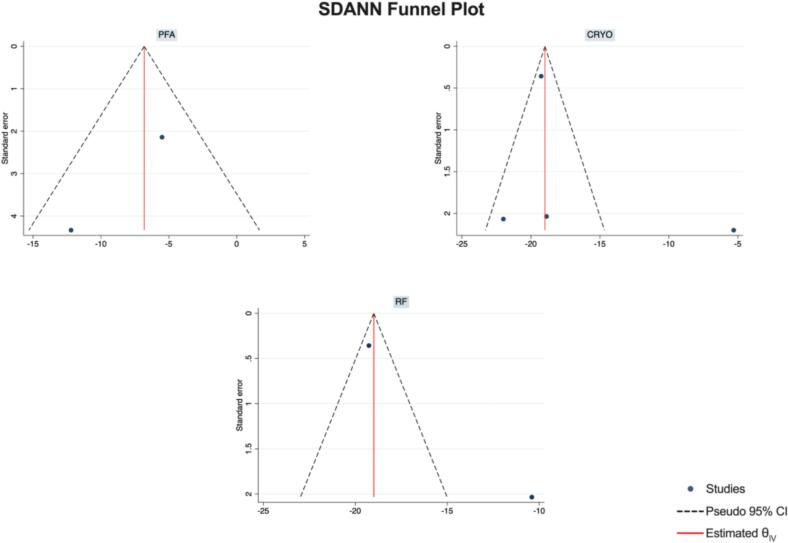
Funnel plots of SDANN.

**Fig. 20:**
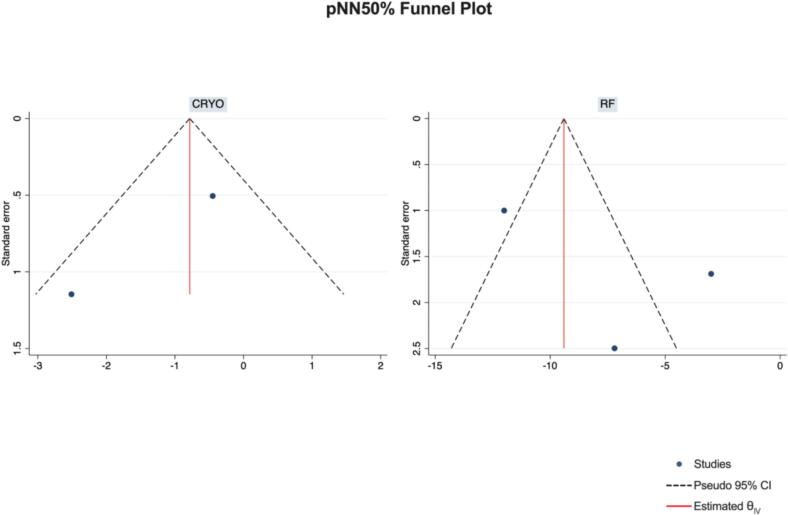
Funnel plots of pNN50%.

**Fig. 21:**
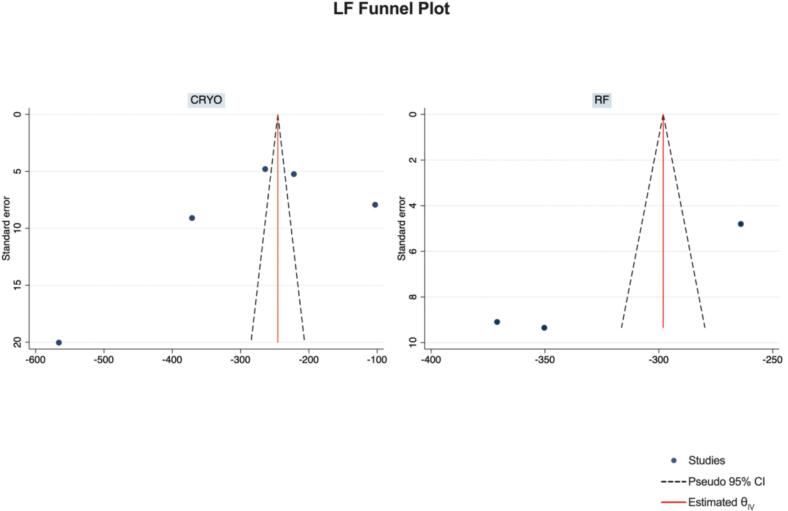
Funnel plots of LF.

**Fig. 22:**
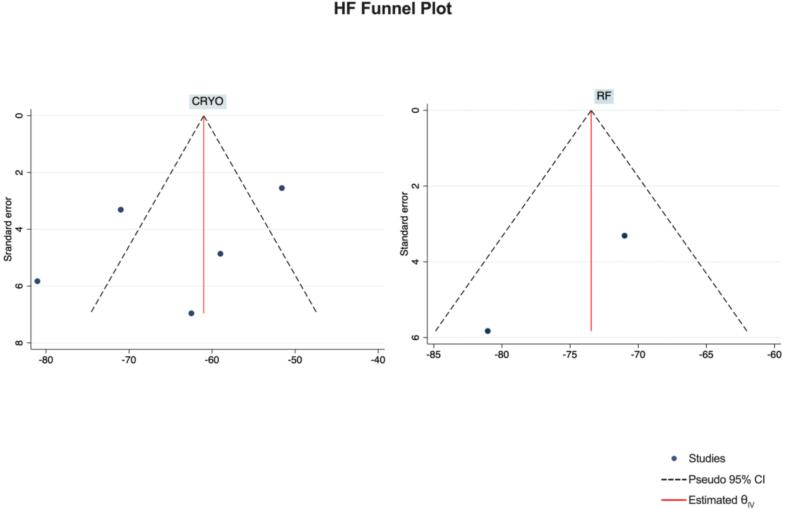
Funnel plots of HF.

**Fig. 23:**
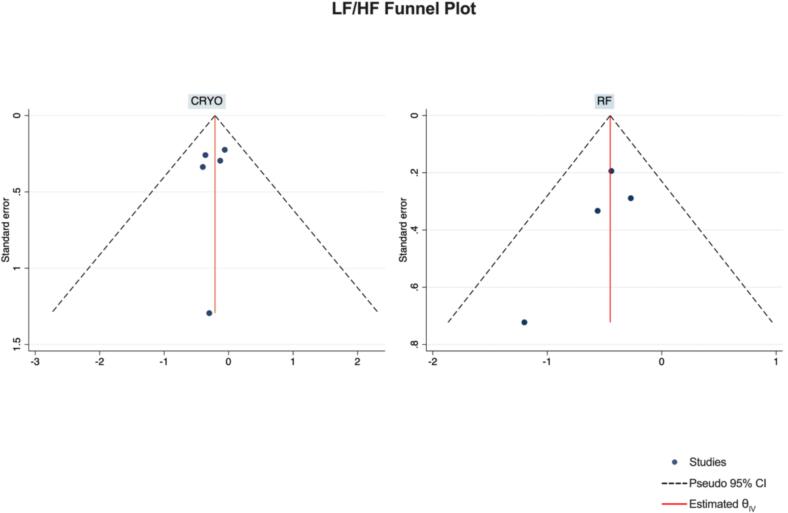
Funnel plots of LF/HF.

In this study, we observed that PFA produced autonomic modifications (Fig. 6, Fig. 7, Fig. 8, Fig. 9, Fig. 10, Fig. 11, Fig. 12, Fig. 13, Fig. 14, Fig. 15, Fig. 16, Fig. 17, Fig. 18, Fig. 19, Fig. 20, Fig. 21, Fig. 22, Fig. 23) in patients with AF, which was found to be less pronounced than CRYO and RF in the subgroup analyses. Furthermore, variations in the autonomic modifying effect were evident across different HRV indices and CA techniques. Further analysis showed that PFA showed a trend toward superior treatment outcomes. Besides, we investigated the differences in the alteration of HRV values between the low-recurrence and high-recurrence subgroups, and the results showed that the changed values of HR, SDNN, and LF/HF differed significantly between the two groups.

These findings underscore the importance of HRV monitoring in managing atrial fibrillation. They also highlight the need for further research to develop more comprehensive therapeutic strategies and improve patient outcomes.

### 4.2 The impact of PFA on cardiac autonomic function

As an integral component of the cardiac autonomic nervous system, ganglionated plexus (GPs) serve as intermediaries for regulating the heart by extracardiac autonomic nerves and independently regulate cardiac function. Meanwhile, parasympathetic nerves predominantly reside within the GPs. [21]. In patients with AF, the abnormally high parasympathetic tone often leads to changes in electrophysiological characteristics, increasing the susceptibility to atrial fibrillation [22]. CA techniques, however, tend to exert an inhibitory parasympathetic effect because their ablation pathways often overlap with the distribution of GPs, particularly around the pulmonary veins [23].

In our study, we observed that PFA exert autonomic modification effects, though demonstrating a weaker autonomic effect than CRYO and RF. PFA is a non-thermal ablation technique that induces irreversible electroporation of cardiac tissues through specific electric field parameters, leading to cell death [24]. A study by Shota et al. demonstrated the relatively mild autonomic impact of PFA, which showed that the release of serum neurological injury markers was lower in patients after PFA [25]. However, the influence of PFA on autonomic function cannot be entirely disregarded. Our study indicates that PFA is associated with increased HR and a significant decrease in SDNN, suggesting a parasympathetic attenuating effect. One potential explanation is that PFA exerts a non-destructive modulating impact on cardiac autonomic nerves, potentially by modulating local neuronal excitability. A study by Guo noted that electrical stimulation might occur during PFA procedures, which could influence the surrounding neural tissue [26]. Meanwhile, an animal study indicates that using PFA to target areas of the heart rich in GPs is feasible and effectively alters markers of cardiac autonomic tone [27].

Additionally, differences were noted among HRV indicators. Specifically, HR and SDANN exhibited more pronounced changes with CRYO, whereas RF affected LF, HF, and the LF/HF ratio more significantly. According to this finding, we can conclude that CRYO exerts a stronger destructive effect on parasympathetic nerves than on sympathetic nerves, although it is weaker compared to RF, which can be attributed to the distinct ablation mechanisms of the two techniques. RF employs high-frequency electrical currents to generate localized heating that affects a broader area, increasing the likelihood of thermal damage to nearby sympathetic and parasympathetic nerves. In contrast, CRYO uses ultra-low temperatures to induce frozen necrosis, providing a more limited impact on surrounding tissues. Since sympathetic and parasympathetic nerves are typically distributed over a certain distance, the cryogenic area produced by PVI may not easily reach sympathetic nerves [28].

### 4.3 The relationship between the impact of PFA on autonomic function and atrial fibrillation recurrence

We also analysed the post-procedural AF recurrence rates associated with the three CA techniques. PFA showed a trend toward more favorable HRV recovery compared to CRYO and RF, though this requires validation in homogeneous RCTs. The effectiveness of PFA in treating AF primarily stems from the isolation of abnormal electrical signals through PVI, which surpasses that of CRYO and RF due to its high ablation efficiency and low contact dependence. A study by Della Rocca et al. demonstrated that PFA could achieve a PVI efficiency of 98.8 %, compared to 81.5 % for CRYO and 73.1 % for RF [29]. Additionally, the low contact dependency of PFA means it does not require as stringent catheter-to-tissue contact as RF and CRYO, allowing for greater procedural flexibility. This feature enables better adaptation to anatomical structures and lesions, improving therapeutic outcomes [30]. However, current evidence from heterogeneous observational studies suggests PFA’s potential to preserve autonomic function, but definitive conclusions await standardised trials.

Considering the strong correlation between autonomic improvement and AF outcomes suggested by numerous studies, future research might explore combining PFA with neuromodulation, such as GP ablation, to achieve enhanced AF outcomes. Meanwhile, although CRYO and RF produce more significant changes in HRV, their clinical efficacy does not match that of PFA. This implies that, for some patients, achieving complete PVI is more crucial than autonomic function attenuation. Vagal denervation may be particularly important for selected patients with vagal-mediated AF. Future studies could focus on this subset of patients to further evaluate treatment efficacy.

We conducted an extensive investigation into the potential correlation between autonomic attenuation and the recurrence profile. Our findings revealed that the low recurrence subgroup demonstrated a more pronounced alteration in HR and SDNN, whereas the degree of alteration in LF/HF was lower. These results suggest that changes in HR and SDNN before and after ablation could predict future recurrence and treatment outcomes. Furthermore, the increased risk of AF recurrence was associated with augmented LF/HF alteration, indicating an enhanced imbalance in the autonomic nervous system by influencing the electrophysiological properties and structural remodeling of the atria.

Notably, the high I^2^ values (exceeding 90 % in several analyses) can be predominantly attributed to the comparison of three distinct ablation techniques. These techniques vary significantly in their underlying mechanisms and procedural protocols, which inherently introduce variability in the outcomes. To mitigate this, we conducted subgroup analyses stratified by ablation type. These analyses revealed a substantial reduction in heterogeneity, with I^2^ values within the PFA subgroup ranging from 0−13.7 %. However, residual heterogeneity persists, primarily due to variations in HRV measurement methods and short − term follow − up durations, which spanned from 1 to 3 months. These findings underscore the importance of standardizing measurement techniques and extending follow-up periods in future studies to minimize such discrepancies.

A discussion of the limitations of this study is warranted. Limitations in existing literature drove the focus on specific HRV metrics, and certain studies lacked comprehensive data on specific HRV measures. Due to gaps in the literature, many potentially essential metrics were not included in the analysis, which may lead to an incomplete assessment of the autonomic nervous system effects. Therefore, future studies should consider a broader array of metrics to fully elucidate the impact of CA techniques on cardiac autonomic nerves. Additionally, the study's temporal focus on alterations within three months post-ablation is a limitation. While the preliminary results provide insights into short-term effects, the long-term implications of these changes remain unclear. The adaptation and recovery of the autonomic nervous system may require a more extended period, particularly in cases involving chronic physiological adaptation and neural remodeling. In addition, although the I^2^ statistic in this study showed heterogeneity in some analyses, it was considered that there were differences in the measurement methods and instruments used by researchers, and that the follow-up time included in this study was within three months, resulting in differences in the measurement time as well.

## 5 Conclusions

A comprehensive *meta*-analysis assessed the impact of PFA on HRV. It revealed a notable decline in HRV metrics following the procedure, with variations observed across different ablation techniques. Additionally, significant disparities were identified in the alterations among HRV metrics. The findings of this study underscore the potential prognostic significance of the autonomic alteration, highlighting the need for further research to develop more comprehensive therapeutic strategies and improve patient outcomes.

## Funding Sources

This work was supported by National Natural Science Foundation of China (82371595), National High Level Hospital Clinical Research Funding from the Chinese Academy of Medical Sciences to Fuwai Hospital (2022-GSP-QZ-4), Chinese Academy of Medical Sciences Innovation Fund for Medical Sciences (2022-I2M-C&T-B-045) and Chinese Academy of Medical Sciences Innovation Fund for Medical Sciences (Grant Number: 2021‐I2M‐1‐063).

## CRediT authorship contribution statement

**Xinyi Wang:** Writing – original draft, Resources, Methodology, Formal analysis. **Zhicheng Hu:** Resources, Methodology, Investigation. **Yan Yao:** Resources, Methodology, Investigation. **Pakezhati Maimaitijiang:** Methodology, Investigation. **Aiyue Chen:** Methodology, Investigation. **Lihui Zheng:** Writing – review & editing, Visualization, Supervision, Funding acquisition.

## Declaration of competing interest

The authors declare that they have no known competing financial interests or personal relationships that could have appeared to influence the work reported in this paper.
